# The polyG diseases: a new disease entity

**DOI:** 10.1186/s40478-022-01383-y

**Published:** 2022-05-31

**Authors:** Tongling Liufu, Yilei Zheng, Jiaxi Yu, Yun Yuan, Zhaoxia Wang, Jianwen Deng, Daojun Hong

**Affiliations:** 1 Department of Neurology, Peking University First Hospital, Beijing, China; 2 Department of Neurology, The First Affiliated Hospital of Nanchang University, Nanchang, China; 3 Beijing Key Laboratory of Neurovascular Disease Discovery, Beijing, China; 4 Department of Medical Genetics, The First Affiliated Hospital of Nanchang University, Nanchang, China

**Keywords:** PolyG diseases, Fragile X-associated tremor/ataxia syndrome, Neuronal intranuclear inclusion disease, Oculopharyngeal myopathy with leukoencephalopathy, Oculopharyngodistal myopathy

## Abstract

Recently, inspired by the similar clinical and pathological features shared with fragile X-associated tremor/ataxia syndrome (FXTAS), abnormal expansion of CGG repeats in the 5’ untranslated region has been found in neuronal intranuclear inclusion disease (NIID), oculopharyngeal myopathy with leukoencephalopathy (OPML), and oculopharyngodistal myopathy (OPDMs). Although the upstream open reading frame has not been elucidated in OPML and OPDMs, polyglycine (polyG) translated by expanded CGG repeats is reported to be as a primary pathogenesis in FXTAS and NIID. Collectively, these findings indicate a new disease entity, the polyG diseases. In this review, we state the common clinical manifestations, pathological features, mechanisms, and potential therapies in these diseases, and provide preliminary opinions about future research in polyG diseases.

## Introduction

Short tandem repeats (STRs) are nucleotide repeats located in both coding and non-coding regions throughout the human genome [103]. STR variants have been reported as the cause of a series of neurodegenerative diseases, including myotonic dystrophies (DM1and DM2), fragile X tremor/ataxia syndrome (FXTAS), some spinocerebellar ataxias (SCAs), and chromosome 9 open reading frame 72 (*C9ORF72*)-related amyotrophic lateral sclerosis (ALS) [21]. More and more studies suggest that STR variants are an important and potential genetic cause of neurodegenerative diseases.

Multiple neurodegenerative diseases have been identified with trinucleotide repeat expansions. The first trinucleotide repeat expansion in a non-coding region on chromosome X was found in patients with fragile X syndrome in 1991 through linkage analysis [114], meanwhile the first trinucleotide repeat expansion in a coding region on androgen receptor gene was identified in Kennedy disease (KD) [56]. With the advances of next-generation sequencing technology, especially in long-read sequencing (LRS), CGG repeat expansion in non-coding regions was found to be associated with a group of previously undetermined late-onset neurodegenerative diseases, including neuronal intranuclear inclusion disease (NIID) [18, 43, 110], oculopharyngeal myopathy with leukoencephalopathy (OPML) [43], and oculopharyngodistal myopathy (OPDM) [19, 43, 129].

The above disorders with non-coding CGG repeat expansion in the 5ʹ untranslated region (5’UTR) share substantial overlap in clinical, neuroimaging, and pathological features, so we hypothesize that they have similar pathogenic mechanisms (Table 1). Polyglycine (polyG) originating from the expanded CGG repeats, which can be translated into a gain-of-function toxic protein, has been reported as an important pathogenic mechanism for FXTAS and NIID [27, 94, 134]. Moreover, preliminary pathological investigations have shown polyG-positive intranuclear inclusions (NIIs) may be deposited in the biopsy specimens from patients with OPDM types 3–4 [127, 129], suggesting a similar pathogenesis with FXTAS and NIID. Accordingly, these disorders have been classified as a novel entity of disease—the polyG diseases [10, 134]. The polyG diseases are a new disease entity, which are characterized by the polyG protein being deposited in the nucleus with a common genetic cause: CGG repeat expansions in the 5’UTR regions.

**Table 1:** Phenotypic features of polyG diseases

	FXTAS	NIID	OPML	OPDM			
				OPDM1	OPDM2	OPDM3	OPDM4
Mode of inheritance	XL	AD	AD	AD	AD	AD	AD
Affected genes	*FMR1*	*NOTCH2NLC*	*NUTM2B-AS1*	*LRP12*	*GIPC1*	*NOTCH2NLC*	*RILPL1*
Gene location	Xq27.3	1q21.2	10q22.3	8q22.3	19p13.12	1q21.2	12q24.31
CGG expansion location	5’UTR	5’UTR	Non-coding Transcript	5’UTR	5’UTR	5’UTR	5’UTR
Physiological repeat numbers	5–50	7–40?	3–16	13–45	12–32	7–40?	9–16
Pathological repeat numbers	55–200	60–300	50–60	85–289	73–164	60–300	139–197
Age of onset (years)	> 50	most > 50	15–40	30–50	20–60	20–50	15–40
*Clinical features*							
Movement disorders	+ + +	+	+	+	+	+	+
Cognitive deficit	+	+ + +	±	−	+	+	−
Autonomic dysfunction	+ + +	+ + +	+	−	−	+	−
Muscle-weakness	+	+	+	+ +	+ +	+ +	+ +
Others	neuropsychosis	encephalitic episodes	myocardiopathy	myocardiopathy	−	−	
*Neuroimage features*							
Brian atrophy	+	+	+	−	−	+	−
Leukoencephalopathy	+	+	+	−	+	+	−
Ribbon sign	±	+	±	−	−	±	−
*Pathology*							
Eosinophilic inclusions	+	+ + +	−	+	+	+	+
Tubulofilamentous inclusion	+	+	−	+	+	+	+
Ubiqutin- or p62- positive	+	+	N/A	+	+	+	+
PolyG-positive	+	+	(polyG)?	(polyG)?	(polyG)?	+	(polyG)?
Rimmed vacuoles in muscle	−	±	−	+	+	+	+

*AD* Autosomal dominant; *XL* X-linked dominant; *AR* Autosomal recessive; *N/A* not available; *UTR* untranslated region

+  +  + , most positive;  +  + , some positive;  + , a few positive;  ± , occasionally positive; −, negative

*FXTAS* fragile X-associated tremor ataxia syndrome; *NIID* neuronal intranuclear inclusion disease; *OPML* oculopharyngeal myopathy with leukoencephalopathy; *OPDM1* oculopharyngodistal myopathy type 1–4; *polyG* polyGlycine peptide toxicity

In this review, we provide a summary of recent findings of polyG diseases, and highlight the similarities and differences among these diseases. The pathogenic mechanism across different polyG diseases, future research directions, potential therapeutic strategies and challenges will be discussed.

## Polyglycine(G) disorders

### Fragile X-associated tremor/ataxia syndrome (FXTAS)

FXTAS is a late-onset, X-linked, neurodegenerative disease characterized by intention tremor, cerebellar ataxia, and cognitive decline, which principally affects males [9, 32]. This disorder is caused by premutation with CGG repeat expansion (55–200 repeats) in the 5’UTR of the fragile X mental retardation 1 (*FMR1*) gene [35]. *FMR1* is located on Xq27.3 and encodes the RNA-binding protein *FMR1* protein (FMRP) [98]. Methylation appears in full mutation CGG expansions (> 200 repeats), leading to transcriptional silencing with consequent deficit FMRP levels in the cells and resulting in fragile X syndrome (FXS), one of the most common inherited forms of intellectual disability and an autism spectrum disorder [23, 89]. Women who carry the expansion will experience lower than 20% risk in having FXTAS due to random inactivation in one of two X chromosomes [34]. Males with CGG repeat numbers less than 71 have low penetrance of FXTAS [71]. In the general population, the prevalence of premutation in CGG repeat expansions is approximately 1 in 300 females and 1 in 850 males [21].

More than one in three adult male premutation carriers present with the neurologic syndrome, including progressive gait ataxia, tremor, cognitive decline, parkinsonism, psychological disorders, and generalized brain atrophy over 50 years of age [35]. Typical neuroimaging changes in FXTAS include symmetrical T2-weighted and fluid attenuated inversion recovery (FLAIR) sequence hyperintense changes in the middle cerebellar peduncle (MCP sign), extensive white matter lesions, brain atrophy, and diffusion-weighted imaging (DWI) sequence hyperintensity at the cortico-medullary junction [45].

Premutation carriers used to be regarded as a phenotypic variant of FXS with normal cognitive abilities for many years. However, recent pathological examination suggested that FXTAS is a different disease entity: a type of inclusion disease with eosinophilic NIIs in both neurons and astrocytes throughout the brain [33]. The NIIs usually contain *FMR1* mRNA, polyG peptides, lamin A and C, ubiquitin, SUMO, and p62 protein, while it is rare for polyglutamine (polyQ) and negative for FMRP or TDP-43 [31]. The NIIs are composed of silk-like substances forming a round and membrane-free structure when viewed under electron microscopy. The NIIs are also found in multiple tissues including the peripheral nerves and skin [112].

### Neuronal intranuclear inclusion disease (NIID)

Neuronal intranuclear inclusion disease (NIID) is a rare progressive neurodegenerative disease caused by non-coding CGG repeat expansions in the *NOTCH2NLC* gene [18, 43, 100, 110]. As suggestive as the name, it is characterized by ubiquitin- or p62-positive extensive eosinophilic NIIs in central and peripheral nervous tissues, which show similar ultrastructural changes as FXTAS under electron microscopy [60, 101]. However, skin biopsy could show NIIs located in the fibroblast, adipocyte, and epithelial cells of sweat gland ducts, which greatly facilitated the antemortem diagnosis of NIID [102]. The clinical features of NIID show great heterogeneity with combinations of cognitive impairments, stroke-like symptoms, encephalitic episodes, autonomic dysfunction, limb weakness, cerebellar ataxia, parkinsonism, peripheral neuropathy, psychiatric disturbance, visual abnormalities, and other multi-system symptoms [57, 119]. The MRI features in adult-onset NIID include diffuse white matter lesions, DWI and FLAIR hyperintensities in the corpus callosum, and DWI linear hyperintensity along the cortico-medullary junction, which are strikingly similar to those of FXTAS [43, 57].

According to the age of onset, NIID can be classified into infantile-onset, juvenile-onset, and adult-onset subgroups [64]. Based on the relationship between phenotype and genotype, researchers divided familial genetically positive adult-onset NIID cases into three subgroups: parkinsonism-dominant NIID, muscle weakness-dominant NIID, and dementia-dominant NIID [110]. Nevertheless, several studies have indicated that carriers with more than 300 repeats of expanded CGG show a mild or asymptomatic phenotype [20, 127]. Beyond NIID, expanded CGG repeats in *NOTCH2NLC* are occasionally related to a small proportion of Parkinson's disease (PD) [65, 97, 110], multiple system atrophy (MSA) [29], essential tremor (ET) [105], degenerative dementia [4, 101], ALS [46, 130], inherited peripheral neuropathy [118], distal motor neuropathy [122, 128], mitochondrial encephalomyopathy, lactic acidosis and stroke-like episodes (MELAS) [57, 123], and oculopharyngodistal myopathy (OPDM) [127]. Intriguingly, CGG expansion in *NOTCH2NLC* was rarely detected in NIID cases reported in people of Caucasian descent, suggesting that NIID is likely to be genetically heterogeneous among different ethnic groups [15].

### Oculopharyngeal myopathy with leukoencephalopathy (OPML)

OPML is an extremely rare genetic disease characterized by ptosis, ophthalmoplegia, dysphagia, dysarthria, and limb muscle weakness [43]. It has only been reported in a four-generation Japanese family, in which seven individuals showed a similar but variable clinical phenotype including severe gastrointestinal dysmotility, respiratory failure, ataxia, bladder disturbances, tremor, and dilated cardiomyopathy. Individuals with diffuse limb weakness underwent muscle biopsies, which showed a nonspecific myopathic change. Cerebral MRI in three affected individuals showed brain atrophy with T2 hyperintensity signals in the white matter consistent with leukoencephalopathy. Intriguingly, one patient showed leukoencephalopathy that was strikingly similar to FXTAS and NIID with characteristic DWI changes at the frontal corticomedullary junctions. Inspired by the common clinical characteristics and neuroimaging features, expanded CGG repeats in whole-genome sequence data were directly explored in one individual from the family. Researchers identified and verified heterozygous trinucleotide repeat expansion CGG in the *NUTM2B-AS1* gene on chromosome 10q22 as the causative gene of OPML.

### Oculopharyngodistal myopathy (OPDM)

OPDM is a rare, adult-onset inherited neuromuscular disorder characterized by progressive ptosis, external ophthalmoplegia, and weakness of the masseter, facial, pharyngeal, and distal limb muscles [26, 93]. Recent studies indicated that CGG repeat expansions in the 5’UTR of the *LRP12* [43], *GIPC1* [19], *NOTCH2NLC* [127], and *RILPL1* [129] genes were associated with OPDM types 1, 2, 3, and 4, respectively. Clinical manifestations of all OPDM subtypes showed a characteristic distribution of muscle involvement. Moreover, a small part of patients with OPDM1/2/4 had extra-muscular symptoms, while most patients with OPDM3 simultaneously had different degrees of leukoencephalopathy, peripheral neuropathy, and other neurological manifestations [82, 127, 128]. The myopathological features were myopathic changes of differing severity characterized by the presence of rimmed vacuoles (RVs) and myeloid bodies in the cytoplasm of muscle cells [19, 82]. NIIs, similar to those of FXTAS and NIID, could be observed in muscle specimens or skin biopsy samples in OPDM1-4, which could facilitate the diagnostic workflow [81].

## The common spectrum of polyG diseases

### Clinical manifestations of polyG diseases

The polyG diseases are adult-onset, slowly progressive, multi-system neurodegenerative disorders that primarily involve the central and peripheral nervous systems and the muscular system. Movement disorder, cognitive disturbance, muscle weakness, and peripheral neuropathy are the common clinical characteristics of polyG diseases.

#### Movement disorder phenotype

Different types of tremor have been described in patients with FXTAS, including ET-like, rest, and cerebellar tremors, and the different tremor types might coexist. In several studies, patients diagnosed with ET were screened for CGG mutations in *FMR1*, *NOTCH2NLC* [105, 125], and *GIPC1* [28], with positive findings in a few patients. Additionally, some OPML patients exhibited mild ataxia or tremor [43]. Collectively, tremors were the most common symptoms of movement disorders in polyG diseases.

Approximately 29% to 60% of FXTAS patients were misdiagnosed as parkinsonism [48, 80, 92]. Parkinsonism is a series of neurodegenerative syndrome characterized by bradykinesia, resting tremor, rigidity, and loss of postural reflexes. Intermediate-length CGG repeat expansion of *NOTCH2NLC* have been associated with PD that is responsive to small doses of levodopa over many years [65, 97]. Expansion of CGG in *GIPC1* was also found in ten out of 1,036 patients diagnosed with PD [28]. In a study of clinical characteristics of *LRP12*-OPDM, one out of 65 patients presented with idiopathic PD in her 70 s [55]**.** Therefore, genetic testing of non-coding CGG repeat expansion in patients with movement disorders of unknown etiology may be helpful for accurate diagnosis and effective treatment.

#### Cognitive deficit phenotypes

Progressive memory loss is a predominant symptom in some patients with FXTAS or NIID [12]. Due to diffuse white matter lesions and DWI hyperintensity along the cortico-medullary junction of frontal-parietal lobes, executive function deficits overlapping with cognitive decline are common symptoms in patients with FXTAS [12] or NIID. Although no cognitive disturbances were reported in OPML patients, extensive white matter lesions and brain atrophy were observed in cerebral MRIs, still the cognitive function of patients was uncertain due to lack of long-term follow-up [43].

The phenotype of OPDMs mainly presents with myopathy and less involvement of the central nervous system (CNS), while a few patients show CNS manifestations [82]. The largest retrospective study of the disorder included a cohort of 64 individuals who were identified as *LRP12* CGG expansion positive, in which 92% showed no significant CNS manifestations [55]. In this study, only one of five patients who underwent biopsy had intranuclear tubulofilamentous inclusions in the bicep muscle with course over eight years. However, a general autopsy was performed in one OPDM1 patient, who had no reported neurological disorder during his lifetime. Unexpectedly, it revealed the presence of NIIs in almost all organs including CNS and peripheral nerves [90]. Approximately 44% of OPDM2 patients with *GIPC1* CGG repeat showed a cognitive deficit [28], and almost all OPDM3 patients had different white matter changes. Collectively, we hypothesized that patients with polyG-related myopathies initially and mainly showed myopathy-based manifestations, while different degrees of cognitive impairment might be unidentified or occur gradually with disease development.

#### Autonomic dysfunction

Autonomic dysfunction is commonly noticed in FXTAS patients, especially impotence and frequent dysfunction of bowel and bladder [33, 45]. Autonomic dysfunction is also prominent and common in patients with adult-onset NIID, and unexplained urinary disturbance is sometimes the only symptom preceding other neurological symptoms for many years [77]. Bilateral miosis is a unique manifestation and diagnostic indicator for NIID [64]. Other autonomic disturbances such as gastrointestinal dysfunction, orthostatic hypotension, arrhythmia, and sexual dysfunction are often recorded in the medical history of NIID patients. Gastrointestinal dysmotility and bladder function have been reported in OPML patients, suggesting the possibility that autonomic nerves are affected by NIIs [43]. Collectively, autonomic dysfunctions in polyG diseases are very common and highly heterogeneous, and can occur more than a decade before typical neuroimaging changes, especially in NIID and FXTAS.

#### Muscle-weakness phenotypes

The names OPDM and OPML indicate that weaknesses of ocular, bulbar, and limb muscles are likely the primary manifestations of these diseases. Additionally, cardiac muscle can be affected, as with dilated cardiomyopathy observed in NIID, OPML and OPDM1 patients [43, 83, 90]. More than half of familial NIID patients showed a muscle weakness-dominant phenotype, which was usually attributed to neurogenic causes, while myopathic origins should not be ignored in some NIID cases. These muscle weakness-dominant patients usually had a younger age at onset, and had a family history associated with larger-size repeat expansions [101]. Similarly, some FXTAS patients may also present with lower limb muscle weakness [45]. The phenotype of muscle weakness in polyG diseases may be associated with NIIs being widespread in nerves and muscles.

### Neuroradiological changes of polyG diseases

**Fig. 1:**
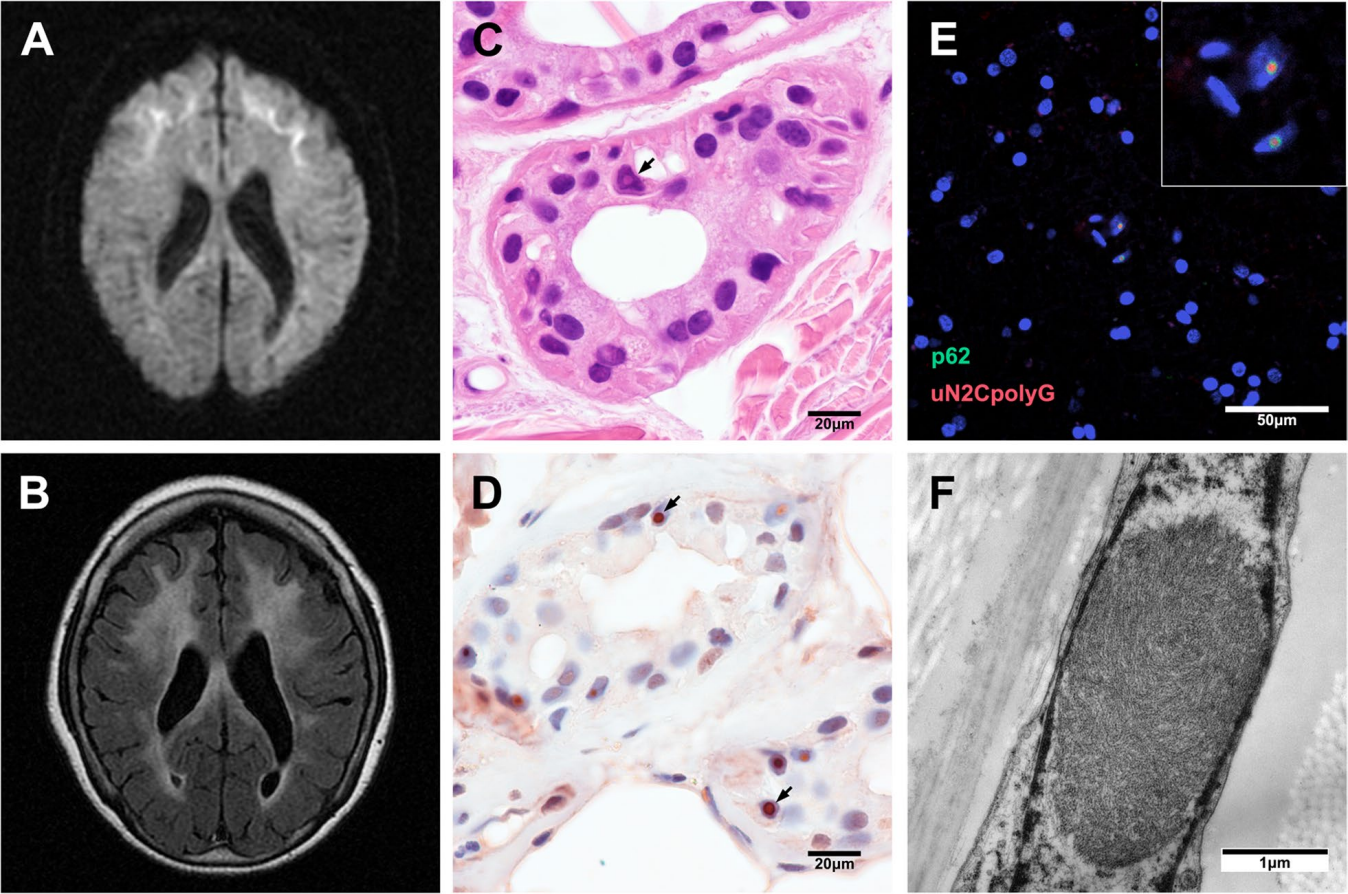
Typical brain radiological and pathological features in patient with *NOTCH2NLC*-CGG expansion. **A and B** Representative brain DWI image and T2-weighted image of patients with *NOTCH2NLC*-related polyG disease. **C** Representative H&E staining image show eosinophilic NIIs (black arrow) in sweat gland cells of skin **D** Representative immunostaining image show p62-positive NIIs (black arrow) in skin. **E** Representative electron microscopy image revealed Intranuclear tubulofilamentous inclusions in the skin of NIID patient. **F** Immunofluorescence against uN2CpolyG using 4D12 antibody (red, generously providing by Dr. Nicolas Charlet-Berguerand) and p62 antibody (green) on brain section from patient with *NOTCH2NLC*-CGG repeat expansion. Nuclei were counterstained with DAPI

Neuroimaging of NIID is a very sensitive and specific biomarker showing that DWI high-intensity signals are distributed along the corticomedullary junction in the frontal and parietal lobes (Fig. 1A). These signals gradually extend along the corticomedullary junction of the whole cerebrum as the disease progresses, but usually do not expand into the deep white matter even late in disease progression [101]. High-intensity subcortical DWI signals in NIID strongly correlate with pathological spongiotic changes of NIID, but the mechanism is still unclear. The “cockscomb pattern” or “ribbon sign” seen along the corticomedullary junction on DWI are characteristic findings in patients with polyG disorders. However, the high-intensity signal at the corticomedullary junction is often not obvious in the early stage of the disease, and can disappear in some patients [49], and thus diagnosis of such diseases should not be overly dependent on the ribbon sign.

White matter hyperintensities and brain atrophy on MRI are other common imaging changes in polyG diseases, including FXTAS, NIID, OPML, and OPMD2-3. The cerebellar involvement in FXTAS is more obvious than cerebrum involvement, and MCP sign and general cortical and subcortical atrophy are seen in the majority of males, and in some women [9, 33]. White matter lesions in NIID and OPML are present in the cerebrum, cerebellum, and spinal cord, and most notably in the deep white matter of the brain (Fig. 1B). Leukoencephalopathy is obvious on MRI in 97.4% of sporadic and 76.9% of familial NIID cases [101]. Additionally, persistent DWI hyperintensities in corpus callosum and enhancement of cortical surface were observed in some NIID cases [57, 119]. More than half of FXTAS patients have cerebral microbleeds, and NIIs in the endothelial cells of capillaries, suggesting cerebrovascular dysfunction in FATXS [91]. Almost all OPDM patients have different white matter hyperintensities but without typical *NOTCH2NLC*-related high-intensity signals at the corticomedullary junction.

### Pathological features of polyG diseases

Each polyG disease has its own pathological features, while the presence of NIIs has been demonstrated in all types of polyG disorders, except for OPML. Eosinophilic NIIs are present throughout the central and peripheral nervous systems, and multiple organs in FXTAS, NIID, and OPDM1 [90]. In contrast, owing to the lack of autopsy histopathological analysis, p62-positive NIIs were only found in biopsied skin and skeletal muscle samples in other types of OPDM patients [129]. The NIIs are round and about 1.5–10 μm in diameter, eosinophilic on hematoxylin–eosin (H&E) staining (Fig. 1C), and positive for p62 and polyG (Fig. 1D and E), and ubiquitin, but negative for polyQ on immunostaining. Under electron microscopy, the inclusions are composed of a pile of fibrous substances often with halos around, but without membranes (Fig. 1F).

Although inclusions are also seen in multiple neurodegenerative diseases such as polyQ diseases [24, 86], frontotemporal dementia (FTLD) [44], OPMD [113, 121] and MSA [84], inclusions in polyG diseases have characteristic immune markers and distribution patterns, suggesting the existence of unique pathological mechanisms. NIIs can be detected more than ten years before the onset of NIID symptoms, and are often found in morphologically intact neurons without obvious neuronal loss [72, 76], suggesting that NIIs may be associated with the progression of the disease. p62-positive rimmed vacuoles in muscle fibers are unique pathological changes in OPDM patients. Muscle biopsy specimens from four OPML patients showed nonspecific myopathic changes [43], but more biopsy specimens are needed to confirm whether OPML has NIIs. Additionally, there is a lack of autopsy results from genetically-positive OPML and OPDM2-3 patients, especially from the CNS. Thus, no direct evidence has demonstrated whether NIIs are present in neurons of OPDM and OPML. Further studies like post-mortem histopathologic analyses are necessary to clarify any extra-muscular organ involvement and associated symptoms that might have been masked by more obvious features relevant to muscle pathology. NIIs involving multiple systems may be responsible for the complex symptoms of polyG diseases.

### Epidemiology and genetics

No prospective longitudinal studies of individuals with polyG diseases have been conducted. Some retrospective studies have been conducted on NIID and FXTAS, which showed significant differences in the population distribution of the two disorders [15, 126]. The reported FXTAS cases were mainly in individuals of Caucasian descent, with a few in Asian populations. Conversely, *NOTCH2NLC*-NIID cases were mainly reported in East Asian populations, with no reports from Caucasian populations. Currently, genetically confirmed cases of OPML and OPDM are mostly from China and Japan, and are rare in Caucasian populations. Whether this difference is related to a founder effect is unclear.

In terms of inheritance, polyG diseases are all dominantly inherited, but many patients with polyG diseases show sporadic pattern with no clear family history. Clinical and genetic anticipation are typical features in most STR-related diseases, while anticipation have not observed in patients with polyG diseases currently. Nucleotide repeat instability and unstable transmission of CGG repeats between parents and offspring have been reported in individual FXTAS, NIID, and OPDM families [5, 20]. These characteristics suggest that polyG diseases may have different molecular genetic mechanisms compared to polyQ diseases.

## Mechanisms

### The puzzle of trinucleotide STR

STRs are a series of small repetitive DNA units consisting of 2–6 nucleotides, covering around 6.77% of the human genome [16]. STRs contribute to genetic diversity, but may be harmful to humans due to repeat-mediated genome instability [37]. Looking back to the past 30 years of studies in repeat expansion disorders [21], polyQ diseases have been characterized by NIIs containing polyQ translated by CAG repeats [59]. Almost 10 years ago, when investigations into the pathogenesis of polyQ diseases was at its beginning stage, one of the seminal questions was how the same mutation present in ORFs of different genes, and thus different proteins with polyQ expansion, could result in different diseases with some common symptoms [79]. Among them, two proteins (androgen receptor and Ataxin-1) with nuclear localization signal (NLS) in two typical polyQ diseases (KD and SCA1) demonstrate the concept that it is the change in normal function and conformation of the polyQ protein, induced by an expanded polyQ tract, which initiates the pathogenic process [54, 85].

In those days, research on the pathogenesis of FXTAS is at a standstill since the repeat expansion is confined to the 5’UTR of *FMR1* [51], despite the fact that CGG repeat expansion is believed to be essential, as said by Ammar Al-Chalabi when the pathogenic locus of *C9ORF72* was uncertain, “It’s like we knew the street, but we didn’t know the exact house” [25]. Fortunately, research on DM1, another disease caused by non-coding trinucleotide repeat expansions, has progressed and demonstrated that repeat sequence-mediated RNA toxicity seems to be involved [117]. Unexpectedly, unlike the typical RNA gain-of-function disease, the majority of the mutant RNA is not located in NIIs, and there is little or no reduction of FMRP in FXTAS [9, 109]. Another hypothesis first proposed by Ranum and colleagues suggested that repeat-associated non-AUG (RAN) translation was involved; this was later confirmed by Todd et al. in FXTAS [111, 136]. Since no or very little FMRpolyA is observed in individuals or disease models of FXTAS, the role of FMRpolyG is an attractive alternative. Furthermore, FMRpolyG is indispensable to the formation of NIIs, as well as mediating the CGG repeat associated toxicity [94]. Recently, FXTAS has its companies that consist of a group of emerging diseases (NIID, OPML, and OPDMs) associated with CGG repeats [135]. PolyG positive NIIs are found in NIID and OPDMs, and expression of polyG by embedding CGG repeat sequence in the 5’UTR of *NOTCH2NLC* is pathogenic in cells and animals. Inspired by polyQ diseases, it led us to infer that polyG is the core pathogenesis of these diseases.

### Repeat-mediated genome instability

Although polyG diseases are reported to be autosomal dominant [21], the hereditary mode is uncertain due to random expansion or contraction of the repeats [52]. For example, offspring of FXTAS patients can be unaffected or suffer from FXS [5]. Unexpectedly, males carrying large CGG repeat expansions (up to 300) in *NOTCH2NLC* seem to be asymptomatic, though *NOTCH2NLC* mRNA levels decrease as a consequence of hypermethylation around the CGG repeats, displaying a strikingly different prognosis in contrast to FXS patients with full mutation alleles of *FMR1* [133]. However, these individuals may have children with NIID if the repeat number is contracted during spermatogenesis [20]. The inconsistent repeat number is related to genome instability, and possibly an explanation of variable disease phenotypes considering the length of polyG tracts translated by the repeats [66].

There are several possible factors underlying repeat-mediated genome instability, including dynamic DNA structures, DNA replication and transcription, DNA repair, and chromatin environment [52]. During DNA replication or transcription, single-stranded DNA can be formed, and the repeat tracts tend to incorporate into different secondary structures determined by the sequence itself. In brief, H-DNA is likely to be formed by homopurine/homopyrimidine mirror repeats [87], G4-DNA by GC-rich sequence, imperfect hairpin by inverted DNA repeat [30], and DNA-unwinding elements by AT-rich sequence [61]. These secondary structures impede replication fork progression and transcription, thus leading to formation of a DNA nick or a double-strand break (DSB), and consequent initiation of DNA repair. The repeat number would undergo unpredictable changes during the process. Moreover, the secondary structures could bring epigenetic changes to the surrounding chromatin, further altering gene expression patterns and repeat instability [104].

For CGG repeats, large repeat length is one of the risks of increasing repeat instability [38]. When located in the lagging strand template during replication, CGG repeats are prone to form hairpins or G4 structures and experience repeat contraction [39, 120]. Large CGG repeats may stall the replication fork, leading to DSBs and chromosome fragility [116, 131]. During DNA transcription, a DNA-RNA hybrid named R-loop is formed on a template strand to regulate gene expression and terminate transcription, leaving the expanding complementary part of the DNA duplex single-stranded to form DNA hairpin (S-loop) [63], G4-DNA or hybrid G4-DNA-RNA structure (G-loop) [2], or a triplex structure of an RNA transcript and a single-stranded DNA portion (H-loop) [78]. These unwanted structures increase the instability of CGG repeats. Additionally, various pathways of DNA repair like homologous replication (HR), end-joining (EJ) pathways, mismatch repair (MMR), base excision repair (BER), and nucleotide excision repair (NER) further increase the risk of genome instability with CGG repeats [52].

### RNA toxicity

RNA-binding proteins (RBPs) are important regulating components of gene expression to support cell viability [7]. The RNA gain-of-function hypothesis was first proposed in DM models based on the observation of nuclear foci containing the myotonic dystrophy protein kinase (*DMPK*) transcript and RBPs interacting with expanded repeats [68, 73, 108], and has become increasingly significant in non-coding repeat expansion disorders. In this hypothesis, RNA foci, generally formed by interactions between secondary structures of repeat RNAs and RBPs, are the hallmark and are found in many repeat expansion diseases, such as amyotrophic lateral sclerosis and frontotemporal dementia caused by *C9ORF72* (C9ALS/FTD), FXTAS, and many SCAs [132]. Though RNA foci are mostly intranuclear, cytoplasmic RNA foci as well as RNA foci at the edge of the nucleus have also been observed [17, 75]. The canonical role of RNA toxicity in pathogenesis is mis-splicing, as confirmed in DM1, CUG repeat RNA sequesters the muscle blind-like (MBNL) proteins and leads to transcriptome-wide spliceopathy [106]. In addition, miRNA misprocessing [87], transcriptional deregulation [11], global translational inhibition mediated by stress granule [41], and alternative polyadenylation sites [6] have also been proposed in recent years.

For FXTAS, the CGG repeat RNA might compromise the function of various RBPs like Pur-alpha, hnRNPA2/B1, CUGBP1, Sam68, and Drosha-DGCR8. Overexpression of most of these proteins can rescue the phenotype in CGG *Drosophila* [47, 95, 96, 99]. However, CGG repeats seem to have a more complex role than could be explained by RNA toxicity alone [94, 111]. Compared with diseases mainly driven by RNA gain-of-function, the pathological repeat range in FXTAS is quite different for the size that is particularly short but close to the pathogenic number of other polyG diseases [21]. Using an RNA FISH probe, CGG repeat RNA formed RNA foci in patients diagnosed with NIID and OPDM types 4. Some RBPs were also found to be colocalized with the RNA foci in polyG diseases like Sam68 and MBNL1 [20, 127, 129]. However, considering the limited role of RNA toxicity in FXTAS, further investigation is needed in other polyG diseases.

### polyG protein toxicity

In most conditions, protein translation is a canonical process beginning with an AUG start codon and ending with one of three stop codons (UAA, UGA, and UAG). However, the AUG-dependent translation initially seemed unable to explain the translation process of CGG expansion embedded within 5’UTR, so RAN translation, an AUG-independent translation, was first proposed by Ranum and colleagues [136]. Based on this hypothesis, RAN translation of CGG repeats in FXTAS can lead to the theorical expression of polyAlanine, polyG and polyArgine containing proteins. However, later studies find that CGG repeats translation still can occur by a canonical ribosome scanning mechanism and initiates upstream of the repeats at near cognate start codons, either a GTG or ACG [50, 94], mainly resulting in expression of one protein, FMRpolyG, as the CGG repeats are in the glycine frame in regard to these GTG or ACG near cognate start codon. Furthermore, antibodies were developed against these putative polyAlanine and polyG proteins and only FMRpolyG was consistently observed in FXTAS brain sections, while the polyAlanine protein was rarely observed or absent [53, 94]. Thus, RAN translation of CGG repeats into polyAlanine or polyArginine is now considered as a minor pathogenic mechanism, if even present, in FXTAS. Similarly, recent reports indicate that translation of the *NOTCH2NLC* CGG repeats into a novel polyG-containing protein occurs through canonical translation initiation at an AUG start codon located upstream of the repeats, with again the CGG repeats in the glycine frame in regards to this AUG start codon[10, 134]. Collectively, both CGG repeat expansion translation in FXTAS and NIID occur through a canonical mechanism of translation, different from the RAN translation proposed by the Ranum group. Intriguingly, an anti-glycine non-specific antibody that may recognize the polyG domain of protein have been found to be colocalized with p62 in NIIs of OPDM3 and 4, indicating a possibility that CGG expansion embedded in the 5’UTR of corresponding genes (*NOTCH2NLC* or *RILPL1*) may be translated into polyG with the same ribosome-dependent translation mechanism [127, 129], but the potential mechanism of OPDM/OPML still needs to be further studied.

Sellier et al. carried out a series of experiments with a fascinating design and provided crucial evidence for discriminating the role of RNA toxicity from protein toxicity [94]. They found that cell models with expanded CGG repeats embedded in the 5’UTR of *FMR1* formed rare RNA foci compared to the expanded CGG repeats without the surrounding *FMR1* sequence. Transgenic mouse models with mutant 5’UTR that express only the CGG RNA remained indistinguishable from control mice. Beyond that, the authors indicated that FMRpolyG first accumulated in the cytoplasm and formed aggregates, and then these aggregates migrated and formed an inclusion within the cell nucleus. Not surprisingly, the phenomenon reappeared in cell models transfected with *NOTCH2NLC* 5’UTR GGC49-GFP (polyG frame), and though there were no fluorescence signals in the cytoplasm, the nuclear polyG inclusions became larger with time. Furthermore, FMRpolyG and uN2CpolyG disrupted both the morphology and function of nuclear lamina, and overexpression of LAP2 rescued neuron death caused by FMRpolyG [134]. According to the above studies in FXTAS and NIID, the significance of polyG in pathogenesis may overwhelm that of toxic RNA, while how much of a role and how polyG plays role in the pathogenesis of polyG diseases remains unclear.

### Future directions for CGG repeat expansion

**Fig. 2:**
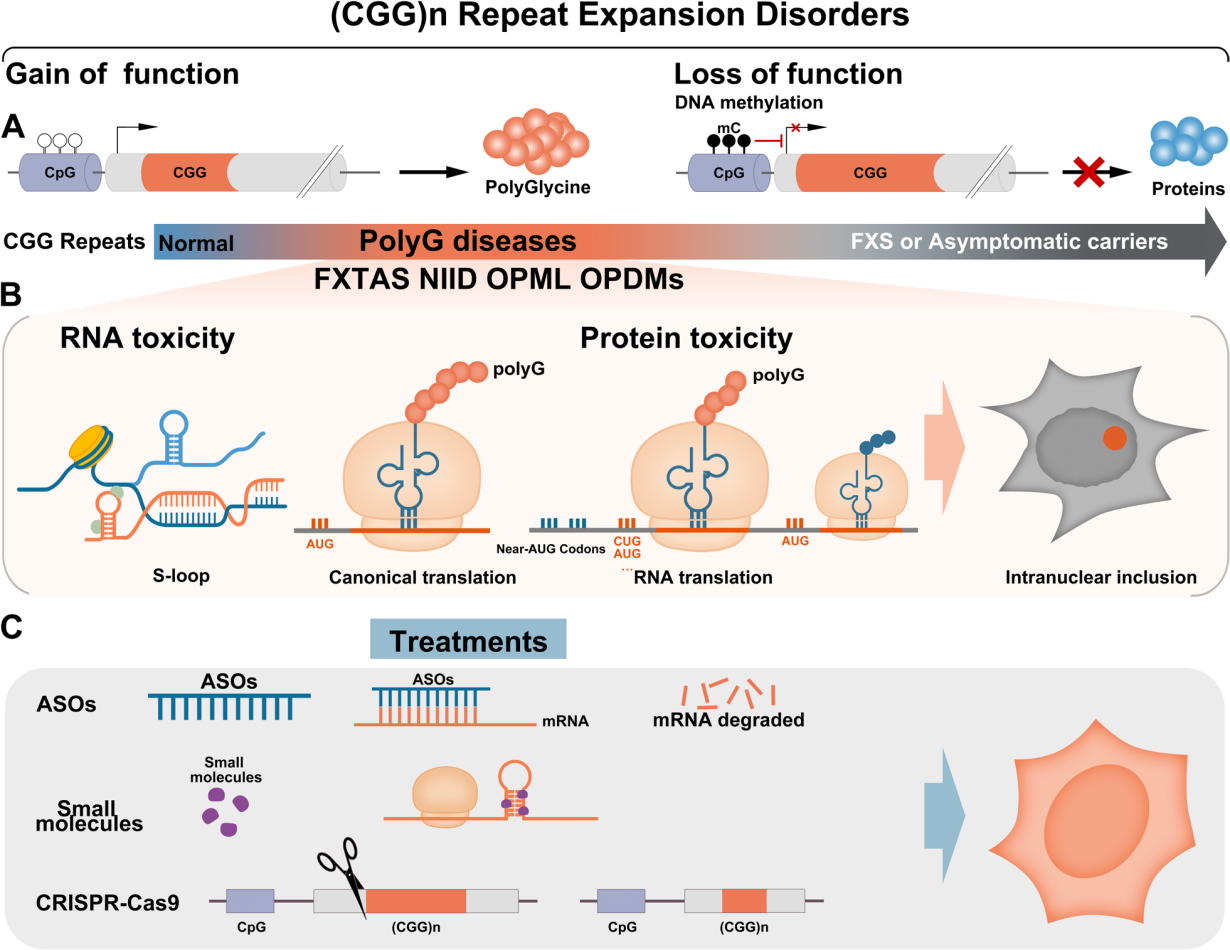
Main mechanisms associated with polyG diseases. **A** CGG repeats are triplet nucleotides located in non-coding regions. There are two main pathogenesis mechanisms of non-coding CGG expansion related diseases. Mild and moderate CGG repeats can translate into polyglycine, and the protein toxicity causes neurodegeneration disorders, including FXTAS and NIID. In contrast, a high degree of repeated CGG sequences will lead to CpG island hypermethylation. Hypermethylation of CpG can lead to transcriptional gene silencing, resulting in partial or complete loss of the native protein encoded by the gene, resulting in abnormal FXS or asymptomatic NIID. **B** Hypotheses for the mechanism of nuclear inclusion body formation. CGG repeat RNAs can fold into complex structures, including hairpins, which aberrantly interact with and sequester RBPs into RNA foci. Non-coding RNA repeats could undergo canonical ribosome-dependent translation mechanism, thereby producing toxic polyG peptides. **C** Possible therapeutic approaches for polyG diseases. ASOs can bind to mRNA that contains pathological repeat expansions, inducing degradation of the target RNA. Small molecules can interact with mutant mRNA and break the hairpin structure. CRISPR/Cas9 technology can be used to excise portions of CGG repeats to inhibit methylation or translation of toxic proteins

Learning from polyQ diseases, we can foresee the productive future of pathogenic mechanism studies in polyG diseases. Here, according to the investigations of polyQ diseases and FXTAS, we would like to give some preliminary opinions about future research in polyG diseases (Fig. 2).

First, what is the role of host genes in pathogenesis? Although CGG repeat expansions may lead us to understand the diseases with a common pathological change, the clinical spectrum is quite varied among polyG diseases [135]. With the same CGG repeat expansion located in non-coding regions and similar repeat-length thresholds for disease penetrance, we wonder if different host genes can partly explain the variety of symptoms. Since the CGG repeat is embedded in uORFs, polyG seems to function independently of the downstream protein expressed by the host genes, while the tissue specific expression pattern of the host gene may limit the toxicity of polyG considering the translation efficiency. Due to separation from the host protein, polyG toxicity is totally different from the mechanism in polyQ that is closely related to the function of the host gene [59]. For example, the way that polyG enters the nucleus is unclear, while polyQ containing protein can be transported to nucleus with NLS expressed by host genes. As shown in previous work, host proteins were partly colocalized with NIIs, especially for *NOTCH2NLC*, the gene which can sometimes be translated into a polyG containing protein [127, 129]. Since CGG repeat translation is initiated upstream of the start codon, it may sequestrate the translation tools and disrupt the expression of downstream proteins [94]. However, the levels of host proteins seem to be unaffected, and individuals remain asymptomatic even once the expression is down-regulated [20].

Second, what is the role of NIIs? NIIs are formed in a series of triplet expansion diseases but their contribution to the disease itself is uncertain [66, 67]. In polyQ diseases, inclusions mainly form in the nucleus, and may be protective in the early phase because they may isolate the toxic polyQ from binding with functional proteins. Inclusions can be toxic when they turn into irreversible solid aggregates [59]. However, long before the NIIs formed, repeat RNA needs to be translated in the cytoplasm and gradually migrate and settle into the nucleus [94]. As indicated in polyQ diseases, the accepted idea is that the nucleus is an important site of polyQ toxicity partly due to the NLS carried by the host proteins [59]. While in polyG-related diseases, when the host proteins no longer work with the toxic tract [10, 94, 134], would the role of nucleus be more like a final location than an active pathogenic site? Like RVs present in OPDMs [90], would polyG reside in other parts of the cell and have a different pathogenic role? Future advancements in disease research may answer these fascinating questions.

Third, what is the role of polyG? As for CGG repeats located in the 5’UTR of *FMR1* and *NOTCH2NLC*, polyG has been identified to be toxic in different disease models, and it may interact with lamin to disturb nuclear function. Overexpression of LAP2B partly rescues the phenotype of FXTAS animal models [10, 94, 134]. This one affected site cannot provide us a whole picture to fully explain the various clinical outcomes, thus more potential targets need to be found in the future. Although recent knowledge about polyG is limited, the future is not so uncertain since polyQ has been found to be correlated with gene expression, axonal transportation, mitochondrial function, neuronal excitotoxicity, and ion homeostasis [43].

## Therapeutic approaches for polyG diseases

There is still a lack of effective treatment for polyG diseases. As more is learned about the pathogenesis of the diseases, several potential therapeutic approaches targeting the DNA, RNA, and protein levels have been developed [124].

Antisense oligonucleotides (ASOs) are chemically synthesized single stranded nucleic acids that can bind RNA targets and prevent them from forming secondary structures and sequestrating the RBPs. Furthermore, the target RNAs can be degraded by RNaseH [8]. ASOs have been successfully applied in treating neurodegenerative diseases caused by repeat expansions. Recent achievements witnessed that ALS fibroblast or induced pluripotent stem cell (iPSC)-derived neurons were rescued by reducing formation of RNA foci and glutamate excitotoxicity [1, 8]. Clinical trials have been conducted in patients with ALS and Huntington's disease (HD) and partly relieve their symptoms [13, 74, 107]. Encouragingly, this kind of therapy has been investigated in models of FXTAS, and specific ASOs greatly improved the clinical and pathological phenotypes in FXTAS mice [22].

Another intervention targeting RNA toxicity is RNA interference (RNAi) strategies, to which purpose is degrading target mRNA and reducing protein expression with RNA molecules including microRNA (miRNA), small interfering RNA (siRNA), and short hairpin RNA (shRNA) arenow the most common RNA molecules for RNAi [14]. For C9ALS/FTD, siRNA effectively reduced GGGGCC repeat-containing transcripts and RNA foci formation in cells and a mouse model [40, 69, 70]. For HD, miRNA has been applied and successfully reduced transcript of huntingtin gene (HTT) levels, leading to improvement in neuropathology [75]. For FXTAS, evidence has shown that using siRNA against RNA of a specific retrotransposon, gypsy, is able to modulate neurodegeneration in *Drosophila* [42].

As for intervention in RNA levels, small molecules can also interact with CGG repeat RNAs. 9-hydroxy-5,11-dimethyl-2-(2-(piperidin-1-yl)ethyl)-6H-pyrido[4, 3-b]carbazol-2-ium, can bind CGG repeats in vitro, improve FXTAS-associated splicing defects, and reduce the size and number of pathologic protein aggregates. Other small molecules identified to interact with CGG repeats include phospholipase A2 inhibitors [88], naphthyridine carbamate dimer [36], piperine, geldanamycin [115], and spironolactone [58].

Repairing DNA levels may be important, but such intervention is risky because it may cause higher repeat associated instability, and thus lead to genome mutations or further expansion of the repeat itself. A study suggests that the expanded repeats can be excised from DNA by CRISPR/Cas9 technology [3]. Expression of *FMR1* can be reactivated by using CRISPR/Cas9 to edit *FMR1* full mutation allele (CGG repeats > 200) in FXS iPSCs [62].

## Conclusions

Four years ago, following the simple but practical principle, with great comparability in phenotypes leading to great comparability in genes, Ishiura et al. [43]. made a breakthrough in STR-related neurodegenerative diseases. They determined that expanded CGG repeats located in non-coding regions were the cause of NIID, OPML, and OPDM type 1. Subsequently, *NOTCH2NLC*-related disorders and other types of OPDM due to abnormal expansion of CGG repeats were reported. Regarding the essential role of polyG in FXTAS and NIID, and polyG-positive NIIs may be also presented in OPDM type 3/4, the diseases caused by CGG repeat expansion can be classified as polyG diseases, highlighting the likely pathogenic role of toxic polyG.

As discussed in this review, the clinical spectrum of polyG diseases is variable, but mainly involves the central and peripheral nervous systems and the muscular system. The disease spectrum may be further enlarged in the future due to the increasing attention on CGG repeat expansion and the advancement of DNA sequencing methods. However, a well-explained polyG-related pathogenesis underlying these different disease phenotypes would be challenging to propose. Until now, the basic research of polyG diseases has focused mainly on modeling the formation and toxicity of polyG in cells and animals, and only one downstream target (nuclear lamina) was discovered [94]. Thus, targeted interventions for the polyG diseases have been stuck on RNAi, gene editing, and small molecules interacting with repeat RNAs, which may lead to genome instability. Future investigations of the diseases may reveal more effective downstream targets of the polyG proteins, and provide us with a more complete picture to better understand, diagnose, and treat the diseases.

## Data Availability

All data generated or analyzed during this study are included in this published article.

## Abbreviations

FXTAS: Fragile X-associated tremor/ataxia syndrome
5’UTR: 5’Untranslated region
NIID: Neuronal intranuclear inclusion disease
OPML: Oculopharyngeal myopathy with leukoencephalopathy
OPDM: Oculopharyngodistal myopathy
uORF: Upstream open reading frame
polyG: Polyglycine
STRs: Short tandem repeats
DM: Myotonic dystrophies
SCAs: Spinocerebellar ataxias
ALS: Amyotrophic lateral sclerosis
KD: Kennedy disease
LRS: Long-read sequencing
NIIs: Intranuclear inclusions
FMR1: Fragile X mental retardation 1
FMRP: FMR1 protein
FXS: Fragile X syndrome
FLAIR: Fluid attenuated inversion recovery
MCP: Middle cerebellar peduncle
DWI: Diffusion-weighted imaging
polyQ: Polyglutamine
PD: Parkinson's disease
MSA: Multiple system atrophy
ET: Essential tremor
MELAS: Mitochondrialencephalomyopathy,lacticacidosis,and stroke-like episodes
RVs: Rimmed vacuoles
CNS: Central nervous system
H&E: Hematoxylin–eosin
FTLD: Frontotemporal dementia
NLS: Nuclear localization signal
RAN: Repeat-associated non-AUG
DSB: Double-strand break
HR: Homologousreplication
EJ: End-joining
MMR: Mismatchrepair
BER: Base excision repair
NER: Nucleotide excision repair
RBPs: RNA-binding proteins
MBNL: Muscleblind-like
FMRpolyG: PolyGlycine translated by CGG repeat transcripts embedded in the 5’UTR of *FMR1*
uN2CpolyG: Polyglycine translated by CGG repeat transcripts in the 5’UTR of NOTCH2NLC
R-loop: DNA-RNA hybrid
S-loop: R-loop with DNA hairpin
G-loop: R-loop with G4-DNA or hybrid G4-DNA-RNA structure
H-loop: R-loop with a triplex structure of an RNA transcript and a single-stranded DNA portion
ASOs: Antisense oligonucleotides
iPSC: Induced pluripotent stem cell
RNAi: RNA interference
miRNA: MicroRNA
siRNA: Small interfering RNA
shRNA: Short hairpin RNA
DMPK: Myotonic dystrophy protein kinase
AD: Alzheimer’s disease
AGD: Argyrophilic grain disease
TDP-43: TAR DNA binding protein 43
